# Depression-associated reductions in putaminal volume are accompanied by a shift from matrix-like to striosome-like structural connectivity

**DOI:** 10.3389/fpsyt.2025.1647240

**Published:** 2025-09-01

**Authors:** An N. Tieu, Jeff L. Waugh

**Affiliations:** ^1^ Division of Pediatric Neurology, Department of Pediatrics, University of Texas Southwestern, Dallas, TX, United States; ^2^ Department of Neurology, University of Texas Southwestern, Dallas, TX, United States

**Keywords:** depression, striatum, striosome and matrix compartments, caudate, putamen, connectivity based parcellation, striosome, matrix

## Abstract

The striatum is among the most studied brain regions in Major Depressive Disorder (MDD) due to its involvement in reward, motivation, and executive functions. The striatum is comprised of interdigitated tissue compartments (matrix and striosome) that have distinct connectivity, pharmacology, and susceptibility to neuropsychiatric diseases. Each compartment is embedded in distinct functional networks, and functional networks that are abnormal in MDD (eg, the default mode and salience networks) selectively couple with the striosome. We hypothesized that imbalances in function between the striatal compartments could correlate with MDD. Historically, assessing compartment-specific involvement in MDD required histological staining of postmortem tissue, precluding *in vivo* or large-group analyses. In a cohort of 266 subjects (MDD and matched healthy controls), we used probabilistic diffusion tractography (connectivity-based parcellation) to identify striatal voxels with matrix-like and striosome-like patterns of connectivity. These compartment-like voxels recapitulated the anatomic features of matrix and striosome described in prior histologic assessments: their relative abundance, location biases within the striatum, somatotopic organization, and distinct patterns of structural connectivity. Prior studies found that decreased putaminal volume was associated with increased risk of developing MDD. We found decreased putaminal volume in MDD, accompanied by a shift from matrix-like to striosome-like volume. We observed an opposing shift in the caudate, from striosome-like to matrix-like volume, that correlated with the severity of MDD symptoms. Our findings suggest that MDD-associated decreased putaminal volume correlates with a shift from matrix-based functional networks toward striosome-dominated patterns in the putamen. Abnormalities of compartment function in the putamen may be a neuroanatomical correlate of the clinical features of MDD.

## 1 Introduction

Major Depressive Disorder (MDD) affects 4% of all adults worldwide (1) and is highly disabling, with long-term disruptions of mood, hedonic, executive, and somatomotor systems (2–4). Despite decades of research and a wide range of therapeutic options, treatment is insufficient for approximately half of MDD patients, underscoring the complex neurological, psychological and social dimensions of the disorder (5–7). Advancing our understanding of potential brain abnormalities associated with MDD, and determining how neuroanatomic differences interact with life factors that trigger or influence depression, may improve future diagnostic and treatment strategies.

MDD has been associated with abnormalities in the striatum, the primary input nucleus of the basal ganglia. Abnormal striatal function, particularly in altered reward processing and motivation, correlates with the symptoms of MDD (8–13). The nuclei that make up the dorsal striatum, the caudate and putamen, are smaller in individuals with MDD (14, 15). In addition, MDD subjects who attempted suicide had decreased gray matter volume in the putamen relative to the control group (16). Functional MRI (fMRI) studies identified abnormalities in striatal networks in MDD, including a doubling of activation area in the frontostriatal salience network (17). In studies of reward anticipation, individuals with MDD had reduced striatal functional activation, and the magnitude of this diminished activity correlated with depression severity (18, 19). Individuals with MDD showed reduced striatal dopamine transporter activity (as measured by positron emission tomography) in the bilateral putamen; whether disrupted dopamine signaling precedes or follows depression is unknown (20). However, in Parkinson disease, higher putaminal dopamine turnover at diagnosis predicted later development of mood disorders (21). These findings highlight that striatal abnormalities are frequently observed in MDD across multiple imaging modalities. However, these findings may be overlooking a critical organizational feature of the striatum: it is comprised of two neurochemically and functionally distinct tissue compartments, the matrix and striosome (22). These compartments are embedded in segregated structural (23, 24) and functional networks (25), suggesting that selectively routing through matrix or striosome may markedly alter information processing and influence on extra-striate regions. Given the structural and functional striatal abnormalities previously observed in MDD, it is essential to learn whether these abnormalities could affect matrix, striosome, or both compartments.

Differences in the structure and function of matrix and striosome may have important implications for compartment-selective vulnerabilities in behavioral and neuropsychiatric disorders. The striosome is a three-dimensional web-like structure that spans the striatum and is interdigitated with and surrounded by the matrix (22). These compartments are distinct in their expression of histochemical markers (26), intra-striate location (27), afferent and efferent connectivity (28), and relative abundance (29, 30). The compartments also exhibit functional differences in animal behavior. Rats will acquire addiction-like behaviors (repetitive lever presses) when stimulating electrodes are located in the striosome, but not in the matrix (31). Preferential stimulation of the striosome also increased pessimistic decision-making behavior in non-human primates (32). In mice, neurons located within the striosome were preferentially activated during reward and punishment learning, relative to neurons located within matrix (33). Dopamine stimulation modulates matrix and striosome in opposite ways, suppressing excitability in the striosome but enhancing it in matrix, enabling rapid shifts in striatal activity (34).

The compartments also show differential vulnerability to degeneration and dysfunction in various neuropsychiatric disorders, such as Huntington disease (HD), Parkinson’s disease, stimulant addiction, and schizophrenia (35–38). Abnormal matrix:striosome volume ratios were previously described in neurodevelopmental and neurodegenerative diseases, such as autism spectrum disorder (39, 40), generalized anxiety disorder, and HD (41, 42). Striosome-specific degeneration has been observed to bias towards specific clinical features in HD: greater degradation of the striosome correlated with a higher burden of mood symptoms (35, 43). Mood symptoms correlate with reduced striosome function in animal models as well; in mice, selective inhibition of the striosome biases approach-avoidance behaviors toward cautious choices, a model of anxiety disorders in humans (44). Given the somatotopic organization of cortico-striate projections, including the somatotopy of projections to matrix and striosome (45), it is likely that the influences of one compartment on neuropsychiatric functions also varies with the part of the cortico-striate circuit being modulated. Likewise, compartment-selective loss of neurons will likely produce different neuropsychiatric abnormalities than compartment-selective changes in neuronal function. Building on evidence that compartment-specific abnormalities can impact neuropsychiatric functions, the goal of the present work was to learn whether shifts in the ratio of matrix:striosome structural networks is associated with MDD.

Historically, it was impossible to study matrix and striosome *in vivo* or at large scale because the compartments could only be identified through histochemical stains in postmortem tissue. We established a diffusion MRI-based method for identifying voxels with matrix-like or striosome-like patterns of structural connectivity *in vivo*, based on decades of injected tract-tracing studies in animals (46). Connectivity-based striatal parcellations are highly reliable, with a test-retest error of 0.14% (46), and highly specific; shifting the position of compartment-like voxels by a few millimeters is sufficient to eliminate any compartment-specific biases in structural or functional connectivity (24, 25). Matrix-like and striosome-like voxels recapitulate the anatomical features of matrix and striosome described in human tissue: their relative abundance, distinct spatial distributions, and biases in structural connectivity (23, 24, 29, 30). We investigated the characteristics of matrix-like and striosome-like voxels in depression, comparing subjects with MDD to matched healthy controls (HC). Individuals with MDD showed smaller matrix-like and larger striosome-like volume in the putamen relative to HC subjects, while compartment-like volumes in the caudate were similar between groups. Matrix-like volume was correlated with depression severity in a specific region within the caudate. These observations suggest that regional abnormalities in compartment bias may correlate with MDD.

## 2 Materials and methods

### 2.1 Experimental cohorts

This was a secondary analysis of MRI and clinical testing data, obtained from either the National Institutes of Mental Health Data Archive (NDA), or ConnectomeDB, the data access portal for the Human Connectome Project (HCP) (47). NDA data can be accessed through this study-specific identifier: 3090. Three studies (shown in **Table 1**) were identified from the NDA based on the availability of high-quality diffusion MRI data and substantial numbers of MDD and HC subjects: Boston Adolescent Neuroimaging of Depression and Anxiety (BANDA) (48){Hubbard, 2020 #2}; Establishing Moderators/Biosignatures of Antidepressant Response (EMBARC) (49) the contribution of aberrant anticipatory processing to spectrum depression and mania, and cognitive and emotional dysfunction in major depressive and bipolar disorders (Aberrant) (50). We created a fourth cohort from the HCP S1200 release (see Supplemental Data for subject identifiers). All imaging and clinical data were de-identified prior to being uploaded to the NDA or ConnectomeDB. All subjects consented to participating in the original studies and consented to anonymously sharing their research data. All research was conducted according to the principles of the Declaration of Helsinki.

**Table 1:** Age, sex, and self-identified race for each study.

Study of origin	Age	Sex	Self-identified race
Aberrant HC: 25 subjects MDD: 25 subjects	Range: 20–43 years Mean: 28 years	HC: 19 female, 6 male MDD: 19 female, 6 male	HC: 3 African American, 4 Asian, 17 White, 1 UK/NR MDD: 3 African American, 3 Asian, 18 White, 1 UK/NR
BANDA HC: 43 subjects MDD: 43 subjects	Range: 14–16 years Mean: 15 years	HC: 30 female, 13 male MDD: 30 female, 13 male	HC: 1 African American, 3 MTOR, 38 White, 1 UK/NR MDD: 7 MTOR, 36 White
EMBARC HC: 31 subjects MDD: 31 subjects	Range: 19–62 years Mean: 37 years	HC: 22 female, 9 male MDD: 20 female, 11 male (65% F)	HC: 6 African American, 1 Asian, 23 White, 1 UK/NR MDD: 6 African American, 1 Asian, 23 White, 1 UK/NR
HCP S1200 HC: 51 subjects MDD: 17 subjects	Range: 22–35 years Mean: 26 years	HC: 15 female, 36 male MDD: 5 female, 12 male	HC: 3 African American, 10 Asian, 2 MTOR, 34 White, 2 UK/NR MDD: 1 African American, 2 Asian, 1 MTOR, 12 White, 1 UK/NR
Combined Cohorts MDD: 116 Subjects HC: 150 Subjects	Range: 14–62 years Mean: 25 years	HC: 86 female, 64 male MDD: 74 female, 42 male	HC: 13 African American, 15 Asian, 5 MTOR, 112 White, 5 UK/NR MDD: 10 African American, 6 Asian, 8 MTOR, 89 White, 3 UK/NR

The racial demographics available for assessment were African American, Asian, MTOR (More Than One Race), UK/NR (Unknown or Not Reported), and White.

Subjects from the NDA were clinically diagnosed with MDD prior to their participation in the original studies. Subjects from the HCP cohort were not previously diagnosed with MDD. Instead, we characterized HCP subjects using the DSM-Oriented Guide for the Achenbach System of Empirically Based Assessment (51). HCP subjects with depression T-scores above 69, the diagnostic threshold for clinical relevance, were classified as MDD subjects. We matched HC to MDD subjects within each originating study, prioritizing in order of importance: sex, age, and self-identified race. For subjects younger than 18 years, we matched age within 12 months. For subjects older than 18 years, we matched within 36 months. The average age of subjects was approximately 25 years for both the MDD group (25.4 years) and HC group (25.7 years). We matched MDD and HC subjects from the NDA studies 1:1. We leveraged the large number of healthy controls in the HCP to generate 1:3 matches (MDD: HC). In total, the combined cohorts included 266 subjects, comprising 116 MDD and 150 HC subjects.

We categorized MDD subjects based on their depression severity using established depression scales. The EMBARC and Aberrant studies used the *Hamilton Depression Rating Scale* (52). The BANDA study used the depression subscale from the *Revised Children’s Anxiety and Depression Scale* (RCADS) (53). Severity categories for the Hamilton scale were: 8-13, Mild Depression; 14-18, Moderate Depression; 19-22, Severe Depression; >22 Very Severe Depression. Severity categories for the RCADS were: 18-20, Mild Depression; 21-23, Moderate Depression; 24-27, Severe Depression; 27-30, Very Severe Depression. For the HCP cohort, we categorized subjects based on the Achenbach T-scores as: 70-77, Mild Depression; 78-85, Moderate Depression; 85-92, Severe Depression; 92-100, Very Severe Depression.

### 2.2 MRI acquisition

Participants from all studies were imaged using 3T whole-brain diffusion tensor imaging (DTI) and T1 (MPRAGE) protocols. Additional scan protocols are available in the studies associated with the originating data (47–50). While some subjects were scanned multiple times in a single study, we only used MRI data from the first time point. The BANDA study consisted of 86 subjects scanned at a DTI resolution of 1.5 mm isotropic. In the EMBARC study, there were 56 subjects scanned at 1.87 x 1.87 x 2.5 mm and 6 subjects scanned at 2.5 mm isotropic. The Aberrant study had 50 subjects scanned at 1.5 mm isotropic. The HCP study consisted of 68 subjects scanned at 1.25 mm isotropic. We matched the study of origin and image resolution when generating MDD: HC matches to account for differences in resolution and image acquisition site.

### 2.3 Diffusion image preprocessing

We processed DTI data using the FSL FMRIB Diffusion Toolbox with standard parameters (54). We performed skull stripping and brain extraction using *bet2* and corrected for eddy current distortion and subject movement during imaging acquisition using *eddy_cuda10.2* (55). We used *dtifit* to assign diffusion tensors to each voxel and *bedpostx_gpu* to model crossing fibers and calculate diffusion probability estimates at each voxel (56). We generated registration matrices from the MNI152_T1 space into FMRIB58_FA diffusion space, and then into native diffusion space using *flirt* and *fnirt*. These registration matrices were used to register all MNI-space regional segmentations into each subject’s native diffusion space. The Aberrant and HCP studies had MRI data scanned in both anterior-posterior (AP) and posterior-anterior (PA) directions, which allowed us to correct for susceptibility artifacts using *topup*. Our other source studies included AP but not PA acquisitions. We performed probabilistic tractography using *probtrackx2* in each subject’s native diffusion space.

### 2.4 Nuclei size and diffusion measures

We performed automated whole-brain segmentation of each subject’s structural (T1-MPRAGE) image using the FreeSurfer toolkit (57), yielding each subject’s estimated total intracranial volume (eTIV). We measured the volumes of the caudate and putamen in native diffusion space using *fslstats* and normalized by the eTIV to control for inter-subject differences in head size. To assess potential tissue injury or maldevelopment, we generated radial diffusivity (RD) by averaging the second and third eigenvalues from the output of *dtifit*. We then extracted RD values using *fslstats*.

### 2.5 Striatal parcellation

We identified striatal voxels with matrix-like or striosome-like structural connectivity using probabilistic diffusion tractography to perform connectivity-based parcellation (58). We created masks of extrastriate target regions that, based on tract-tracing studies in animals and/or diffusion MRI in humans, selectively project to either the matrix or striosome compartments (46). Specifically, we generated matrix-favoring and striosome-favoring composite “bait” masks by summing regions whose structural connectivity was biased toward either the matrix (inferior frontal gyrus pars opercularis, primary motor cortex, supplementary motor area, primary somatosensory cortex, and superior parietal cortex) or the striosome (posterior orbitofrontal cortex, anterior insula, basolateral amygdala, basal operculum, and posterior temporal fusiform cortex). We previously utilized these regions in a separate MRI dataset to map compartment-like connectivity (23). We used composite bait regions for each compartment because while injected tract tracers in animals identified compartment-favoring biases, these were not absolute: regions could still include voxels with connectivity to the other compartment (59). Similarly, a region may favor one compartment within a specific striatal somatotopic zone but lose that bias in other striatal areas (25, 46, 60). By using summed bait masks, we minimized these sources of somatotopic variability when assessing compartment-like bias. The striatal mask included the caudate and putamen but excluded the posterior half of the caudate tail, as this region contains very little striosome (61) and its narrow structure in the coronal plane reduced registration accuracy and led to partial volume effects with the surrounding white matter. We excluded the nucleus accumbens due to the absence of prior tract-tracing studies to guide the selection of matrix- and striosome-favoring bait regions. The diffusion voxels in our study were larger than the branches of the human striosome (maximum cross-sectional diameter: 1.25 mm (29, 30), so even highly biased striosome-like voxels included some matrix tissue. Highly biased matrix-like voxels, in contrast, could include only matrix tissue given that the matrix compartment is both substantially larger and likely to occur in segregated parts of the striatum. We used the terms “striosome-like” or “matrix-like” to remind readers that these are indirect and probabilistic parcellations that should not be equated with matrix and striosome identified through the gold standard for tissue-level parcellation, histochemical staining.

We performed striatal parcellation using FSL’s *probtrackx2* in classification targets mode, setting each striatal voxel as a seed and the composite compartment-favoring bait region masks as targets. *Probtrackx2* used the following parameters: curvature threshold=0.2; step length=0.5 mm; 2,000 steps per sample; 5,000 streamlines per seed voxel. To prevent mis-tracked interhemispheric streamlines, we parcellated each hemisphere separately, using a contra-hemispheric exclusion mask. All tractography was performed in the subject’s native DTI space.

We utilized the FSL tool *proj_thresh* to calculate the voxelwise probability of connecting to matrix-favoring or striosome-favoring target masks. This approach generates two superimposable striatal probability maps, one for the matrix-like and one for the striosome-like distribution, each with P=0-1. At each striatal voxel, the sum of these two probabilities was always one. Within each distribution we defined voxels with P≥0.55 as reaching bias threshold, and thus meeting criteria to be defined as matrix-like or striosome-like. Voxels between the lower limits of the compartment-like distributions (P=0.45-0.55) were defined as indeterminate. Across the full range of thresholds (0.55<P<1.0), the matrix-like and striosome-like voxels consistently replicate known compartment tissue characteristics from histological literature, such as relative abundance and spatial distribution. A threshold of P>0.55 quantifies all biased voxels for the purposes of performing group level comparisons, while a stricter threshold of P>0.87 can be used for targeting voxels with stronger compartment-like bias – and therefore, a purer reflection of the properties of one compartment. We quantified the volume of matrix-like and striosome-like voxels separately in caudate and putamen. We used *fsl-cluster* to measure the size of the largest matrix-like and striosome-like clusters.

### 2.6 Generating high-bias compartment-specific masks

In animal and human tissue, the ratio of striosome:matrix cross-sectional area is approximately 15:85 (62–64). Our MRI-based striatal parcellations, in other human datasets, produced similar ratios (23, 25, 40, 46). While analyses that include all compartment-like voxels are useful for some measures, the natural differences in size between the compartments can bias probabilistic measures, such as tractography. Since histology predicts that matrix-like volume will be approximately 5-fold larger than striosome-like volume, we created equal-volume masks from the most-biased voxels from each distribution. We set the target size for these masks at 13% of total striatal volume: below the expected lower limit for striosome-like volume, and equal to the volume 1.5 standard deviations above the mean in a normal distribution. To account for differences in diffusion MRI resolution between the studies, the ratio of compartment-like volume to striatum volume was kept constant between studies. For cases in which one compartment’s distribution was smaller than the target volume, we equalized inter-compartment volume by reducing the size of the larger compartment mask to match that of the smaller compartment mask; the volume of these high-bias matrix-like and striosome-like voxels was always equal. We extracted the cartesian location of each voxel in these high-bias masks relative to the centroid of its nucleus of origin (caudate or putamen). We measured RD within each compartment’s high-bias mask.

### 2.7 Inverse seed tractography between compartment-like voxels and bait regions

To assess the robustness and specificity of structural connectivity between compartment-like voxels and their corresponding bait regions, we performed inverse seed-to-target tractography between compartment-like voxels and their associated bait regions. We performed both A-to-B and B-to-A streamline tractography, first setting compartment-like voxels as seeds and bait regions as targets, then setting the composite bait region masks as seeds and compartment-like voxels as targets. We used the same *probtrackx2* parameters described above for striatal parcellation (Methods 2.5), save for the use of streamline mode rather than classification targets mode. We used *waypoint* and *avoid* flags to include one compartment-specific target and exclude the other. We normalized the streamline counts to the volume of the seed mask to account for the marked size differences in bait region and compartment-like masks.

### 2.8 Somatotopic organization of corticostriate projections

To quantify the influence of each bait region on compartment-like bias, we performed 10 subsequent rounds of probabilistic tractography, each using only nine bait regions, rotating the left-out bait region for each round. These 10 “N-1” parcellations allowed us to quantify connectivity with the omitted region and generate masks identifying the discrete areas most-influenced by each bait region. We subtracted each “N-1” parcellation from the original (all bait regions) parcellation; the differences between these parcellations quantified the influence of the left-out bait region at a voxelwise level. We thresholded these difference maps (using *fslmaths*) to ensure that each somatotopic zone of influence was distinct, with no overlap between other bait regions’ somatotopic zones, and that each somatotopic zone was similarly sized. We then quantified compartment-like volumes (P≥0.55) within each of these 10 somatotopic zones. Volumes were presented as a ratio of compartment-like volume differences which allowed us to represent changes in both compartments as a single data point. In zones influenced by matrix-favoring bait regions we assessed region-specific volume as $\frac{Vol\left(Matrix\right)\;-\;Vol\left(Striosome\right)}{Vol\left(Matrix\right)\;+\;Vol\left(Striosome\right)}$ and for zones influenced by striosome-favoring bait regions as $\frac{Vol\left(Striosome\right)\;-\;Vol\left(Matrix\right)}{Vol\left(Matrix\right)\;+\;Vol\left(Striosome\right)}$ .

### 2.9 Statistical analyses

We performed a series of ANOVAs to test for significant group differences between MDD and HC subjects in nuclei size, compartment-like volumes, and radial diffusivity. Scanner type, study of origin, and age were included as covariates of no interest to control for potential confounding effects. We corrected for multiple comparisons using the family-wise error (FWE) method of Benjamini and Hochberg (65). We defined the following families of tests for these FWE corrections: Nuclei Size (2 tests), Radial diffusivity of nuclei (2 tests), Compartment-like volumes between MDD and HC groups (4 tests), Radial diffusivity of high-bias voxels (2 tests), Compartment-like volume varying by severity in the caudate (2 tests), Somatotopic zone compartment-like volumes (10 tests).

We performed two-tailed, two-sample t-tests with unequal variance to assess several approaches to validating our striatal parcellations: the mean location of high-bias compartment masks, compartment cluster size, and streamline counts in the inverse seed-to-target analysis. We defined the following families of tests for these FWE corrections: High-bias compartment masks locations (3 tests), Compartment cluster size (2 tests), Inverse seed streamline counts (4 tests).

In addition to these family-specific t-tests, we used the FSL tool *randomise* to perform voxel-wise nonparametric permutation inference testing of the whole striatum to identify voxels with significant differences in compartment-like bias between MDD and HC. We performed a second iteration of *randomise* to test if compartment-like biases correlated with depression severity, solely within our MDD cohort. While we generated tractography in each hemisphere independently, we combined the hemispheres for each subject prior to *randomise* testing to reduce the number of comparisons. We performed *randomise* with 5,000 permutations, 2 mm variance smoothing, threshold-free cluster enhancement, and masked by the striatum. We calculated the center of gravity and size for each significant cluster from *randomise* using *fslstats*. For testing MDD vs. HC group level differences and MDD severity effects, we included study-of-origin, scanner, and age as covariates of no interest within *randomise*.

## 3 Results

### 3.1 Group differences in nuclei volume and radial diffusivity

MDD subjects exhibited a lower total intracranial volume (eTIV) relative to healthy control subjects (MDD: 1381 cm^3^ vs. HC: 1438 cm^3^; F_1,531_, p = 0.048). We then compared volumes of the caudate and putamen, normalized to eTIV, between MDD and HC subjects. The normalized putamen volume in MDD subjects was 3.7% smaller (F_1,531_, p = 3.4x10^-4^), a pattern that was also observed in raw putamen volume (MDD: 3857 cm^3^ vs. HC: 4207 cm^3^, 8.3% smaller; F_1,531_, p< 1.0x10^-6^). In contrast, normalized volume differences in the caudate were nonsignificant between groups, showing a 5.6% increase in MDD (F_1,531_, p = 0.089). Raw caudate volumes also exhibited no significant difference between groups (MDD: 3235 mm^3^ vs. HC: 3246 mm^3^; F_1,531_, p = 0.73). We compared radial diffusivity (RD) between MDD and HC subjects for the caudate and putamen. RD was increased by 4.7% in the MDD putamen but only trended towards significance (MDD: 5.1x10^–4^ vs. HC: 4.9x10^-4^; F_1,531_, p = 0.027) after multiple comparisons correction. Caudate RD differences were nonsignificant (MDD: 6.2x10^–4^ vs. HC: 5.8x10^-4^; F_1,531_, p = 0.39).

### 3.2 Compartment clusters

In histologic sections, the striosome appears as separated “islands” while the matrix is a contiguous structure (27, 30). If connectivity-based striatal parcellations truly approximate the organization of matrix and striosome, compartment-like voxels should follow this schema (contiguous vs. separated). We used *fsl-cluster* to assess the volumes of the largest matrix-like and striosome-like clusters at a compartment-like probability bias of P>0.55. Matrix-like voxels were far more likely to occur in large, contiguous clusters; the mean volume of the largest cluster of matrix-like voxels was 571 mm^3^, while the largest striosome-like cluster was 162 mm^3^, a 3.5-fold difference (p = 6.3x10^-101^). This indicates that matrix-like and striosome-like voxels followed the same organizational schema (contiguity vs. separation) seen in matrix and striosome tissue.

### 3.3 Location of high-bias compartment-like voxels

To demonstrate the similarities between compartment-like voxels and compartment characteristics derived from histology, we extracted the location of each voxel in our high-bias compartment masks relative to the centroid of its corresponding nucleus (caudate or putamen). Striosome-like voxels were, on average, located within 1 mm of matrix-like voxels in the medial-lateral plane, but were positioned 5.5 mm more rostral (p<1.0x10^-100^) and 5.6 mm more ventral (p<1.0x10^-100^) than matrix-like voxels. The mean location of matrix-like and striosome-like voxels mirrored the compartment location biases found in human histology (27, 30).

### 3.4 Inverse seed tractography between compartment-like voxels and bait regions

**Figure 1:**
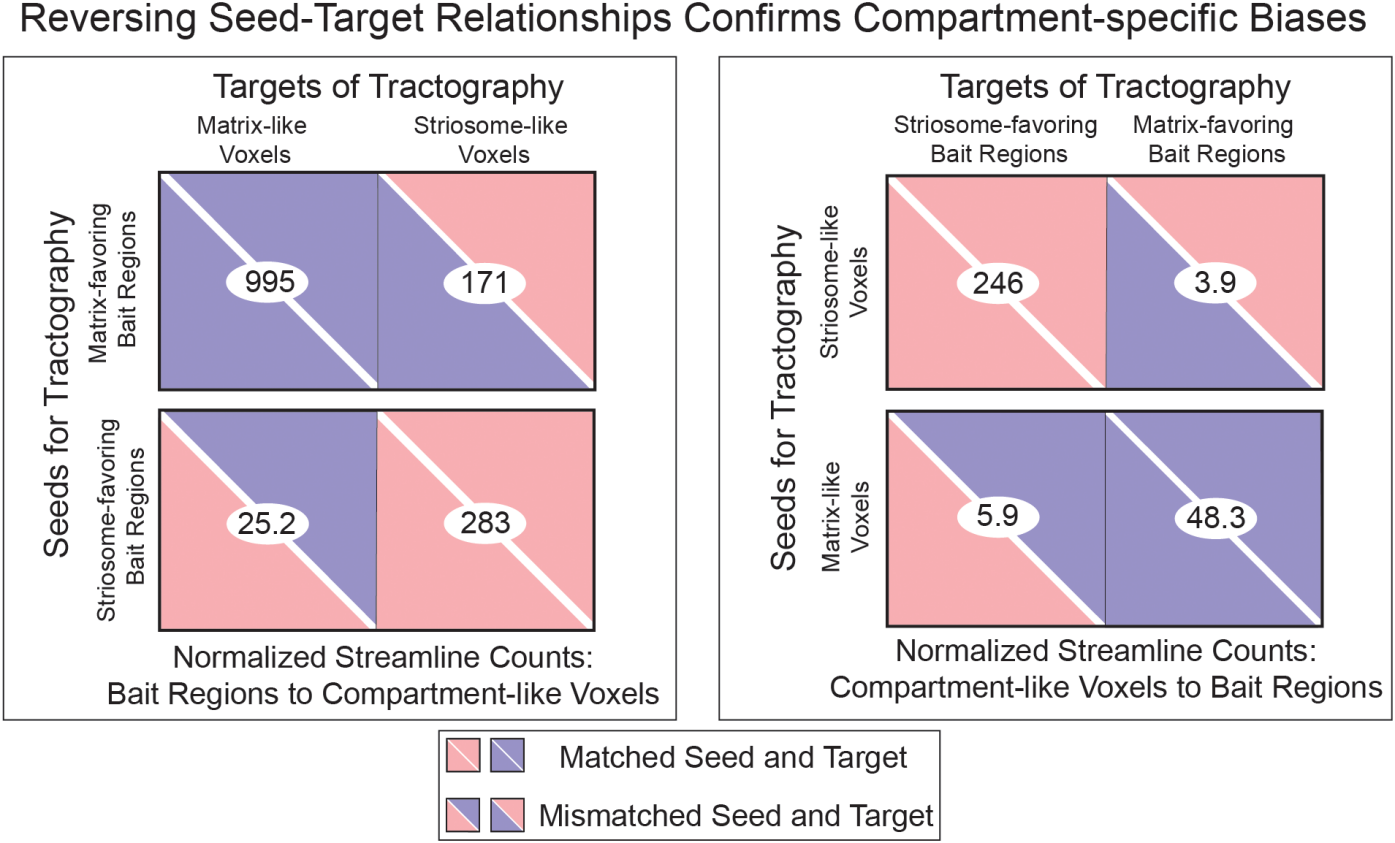
Compartment-like voxels and their corresponding bait regions are biased to each other in tractography. Streamline counts from compartment-like voxel-to-bait regions (A-to-B) and bait regions-to-compartment-like voxel (B-to-A) tractography demonstrated the same pattern: matched seeds and targets always had significantly greater streamline counts than mismatched seeds and targets. Matrix-like voxels exhibited stronger connectivity with matrix bait regions than with striosome bait regions, and similarly, striosome-like voxels showed stronger connectivity with striosome bait regions than with matrix bait regions. This pattern is consistent for both seed-to-target (A-to-B, B-to-A) variations. Streamline counts were normalized to the size of the seed volume. All streamline comparisons (matched vs. mismatched, paired samples t-tests) were significant (p< 1.0x10^-33^) following family-wise error correction.

Our striatal parcellations were executed in classification targets mode, which quantified connectivity between individual striatal voxels and the bait region targets. As a further validation of the connectivity biases underpinning these parcellations, we carried out streamline tractography with compartment-like voxels as seeds and the compartment-favoring bait masks as targets. We then inverted seed and target (composite bait region masks as seeds, compartment-like voxels as targets), allowing us to assess bias in two modes (A-to-B, and B-to-A). Streamlines-per-seed followed the biases predicted from animal tract tracing studies (46) and by our connectivity-based parcellations. Normalized streamline counts were significantly larger when the compartments were matched (striosome-to-striosome, matrix-to-matrix) than when they targeted the opposing compartment (**Figure 1**). This was true with both striatal compartment-like voxels as seeds, and when we set compartment-favoring bait regions as seeds. Normalized streamline counts were significantly different when seeds and targets were matched vs. unmatched. Specifically, matrix-favoring bait regions routed more streamlines to matrix-like voxels (995) than to striosome-like voxels (171; p = 4.2x10^-35^). Striosome-favoring bait regions similarly projected more to striosome-like voxels (283) than to matrix-like voxels (25.2; p = 4.1x10^-37^). When using compartment-like voxels as seeds, matrix-like voxels sent more streamlines to matrix-favoring bait regions (246) than to striosome-favoring bait regions (3.92; p = 4.1x10^-37^). Striosome-like voxels sent more streamlines to striosome-favoring bait regions (48.3) than to matrix-favoring bait regions (48.3; p = 8.0x10^-29^).

### 3.5 Compartment volumes, MDD vs. HC

**Figure 2:**
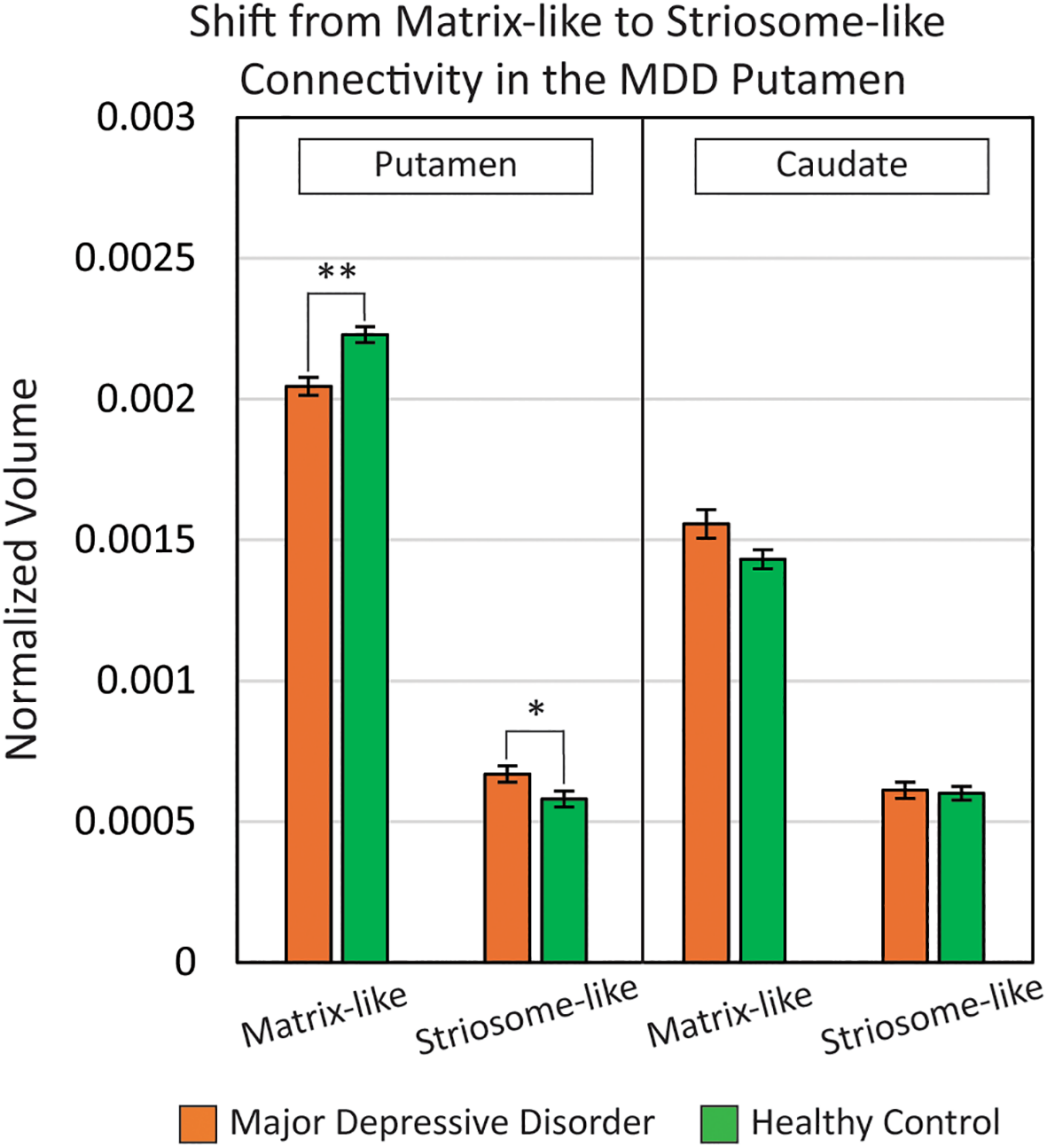
In the putamen of major depressive disorder (MDD) subjects, there was a shift from matrix-like volume to striosome-like volume (matrix: -8.2%; striosome: +15%). Compartment-like volume differences in the caudate were nonsignificant between MDD and healthy control groups. Compartment-like volumes were normalized to each subject’s total intracranial volume. **p = 2.3x10^-8^; *p = 9.0x10^-3^. Results are FWE-corrected for multiple comparisons.

We compared compartment-like volumes in the caudate and putamen, normalized by eTIV, between MDD and HC groups (**Figure 2**). In the MDD putamen, normalized matrix-like volume was 8.2% smaller (F_1,531_, p = 2.25x10^-8^) while normalized striosome-like volume was 15% larger (F_1,531_, p = 9.0x10^-3^). Raw volume measures in the putamen also exhibited this shift from matrix-like to striosome-like volume: Matrix-like (MDD: 2785 mm^3^ vs. HC: 3197 mm^3^; F_1,531_, p = 3.9x10^-9^); Striosome-like (MDD: 857 mm^3^ vs. HC: 757 mm^3^; F_1,531_, p = 0.021).

In the caudate, there were no significant differences in normalized compartment-like volume between MDD and HC subjects. In MDD, normalized matrix-like volume in the caudate was 8.7% larger (F_1,531_, p = 0.31) while normalized striosome-like volume was 1.7% larger (F_1,531_, p = 0.42). Raw volume differences between groups were also nonsignificant in both matrix-like and striosome-like voxels: Matrix-like (MDD: 2075 mm^3^ vs. HC: 2025 mm^3^; F_1,531_, p = 0.55); Striosome-like (MDD: 2075 mm^3^ vs. HC: 2025 mm^3^; F_1,531_, p = 0.55).

### 3.6 Compartment specific RD, MDD vs. HC

We analyzed RD within our high-bias compartment-like masks to investigate whether abnormalities in compartment-like volume might be attributable to compartment-specific injury or maldevelopment. When we removed the influence of age, sex, and scanner type, we found no significant differences in RD between MDD and HC groups in either matrix-like or striosome-like voxels: Matrix-like (MDD: 5.5x10^–4^ vs. HC: 5.0x10^-4^; F_1,531_, p = 0.65); Striosome-like (MDD: 6.0x10^–4^ vs. HC: 5.3x10^-4^; F_1,531_, p = 0.14).

### 3.7 Voxels with significant compartment-bias differences, MDD vs. HC

**Figure 3:**
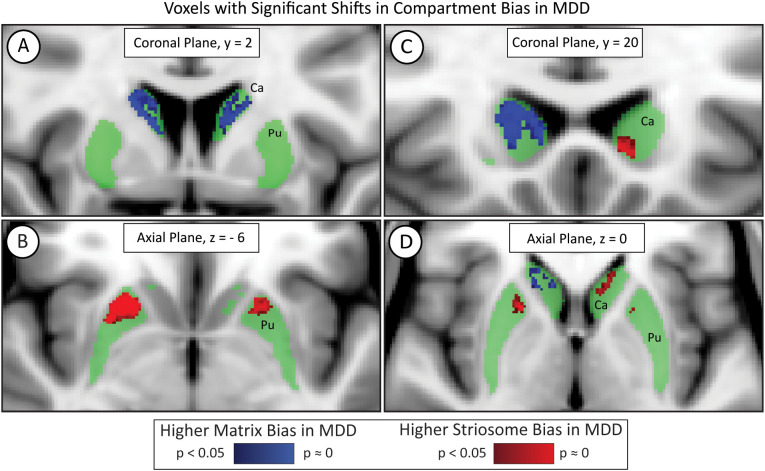
Voxelwise testing with FSL’s *randomise* identified significant differences in compartment-like bias in major depressive disorder (MDD). While most of the striatum (green) did not differ in MDD, voxels in the bilateral rostral putamen (Pu) were significantly shifted toward striosome-like bias (red; **B**) in MDD subjects. The caudate (Ca) had a region of increased matrix-like biased (blue; **A**) but also exhibited laterality effects. The left caudate demonstrated an increased striosome-like bias (red; **C, D**), while the right caudate showed a substantially larger increase in matrix-like bias (blue; **A, C, D**). Significance threshold, p< 0.05, FWE-corrected within *randomise*. The images adhere to radiographic convention and coordinates follow MNI convention.

We identified clusters of voxels with significant differences between MDD and HC subjects in compartment-like bias at a voxelwise level using FSL’s nonparametric inference testing utility, *randomise* (**Figure 3**). Because each voxel’s compartment-like biases always sum to one, changes in the amplitude of matrix-like bias are always countered by a comparable shift in striosome-like bias. Therefore, we included the full probability distribution (P=0-1) of the matrix-like striatal probability maps, thereby assessing both compartments in one iteration of *randomise*.

In the bilateral rostral putamen, the connectivity bias in MDD subjects shifted from matrix-like toward striosome-like bias. The volume of putaminal voxels with significantly shifted bias in MDD was 236 mm³ in the left hemisphere and 683 mm³ in the right hemisphere. The left and right putaminal clusters had highly symmetrical centers of gravity (COG); the MNI coordinates (x-, y-, z-planes) for the left significant cluster were (11, 21, 5) while those for the right were (11, 22, 4).

In the caudate, the left and right hemispheres exhibited opposing abnormalities in compartment-like bias. The right hemisphere in MDD had a large cluster of voxels in which bias was shifted from striosome-like toward matrix-like (1834 mm³), while the left hemisphere had a small volume in which voxels shifted from matrix-like toward striosome-like bias (116 mm³). These significant clusters were located similarly in the x and y planes but differed substantially in the z (axial) plane: left COG (10, 17, 1) vs. right COG (11, 11, 13). Notably, these interhemispheric differences in bias may explain why our measures of caudate compartment-like volume (Section 3.5) did not differ between MDD and HC.

### 3.8 Compartment biases correlate with MDD severity

**Figure 4:**
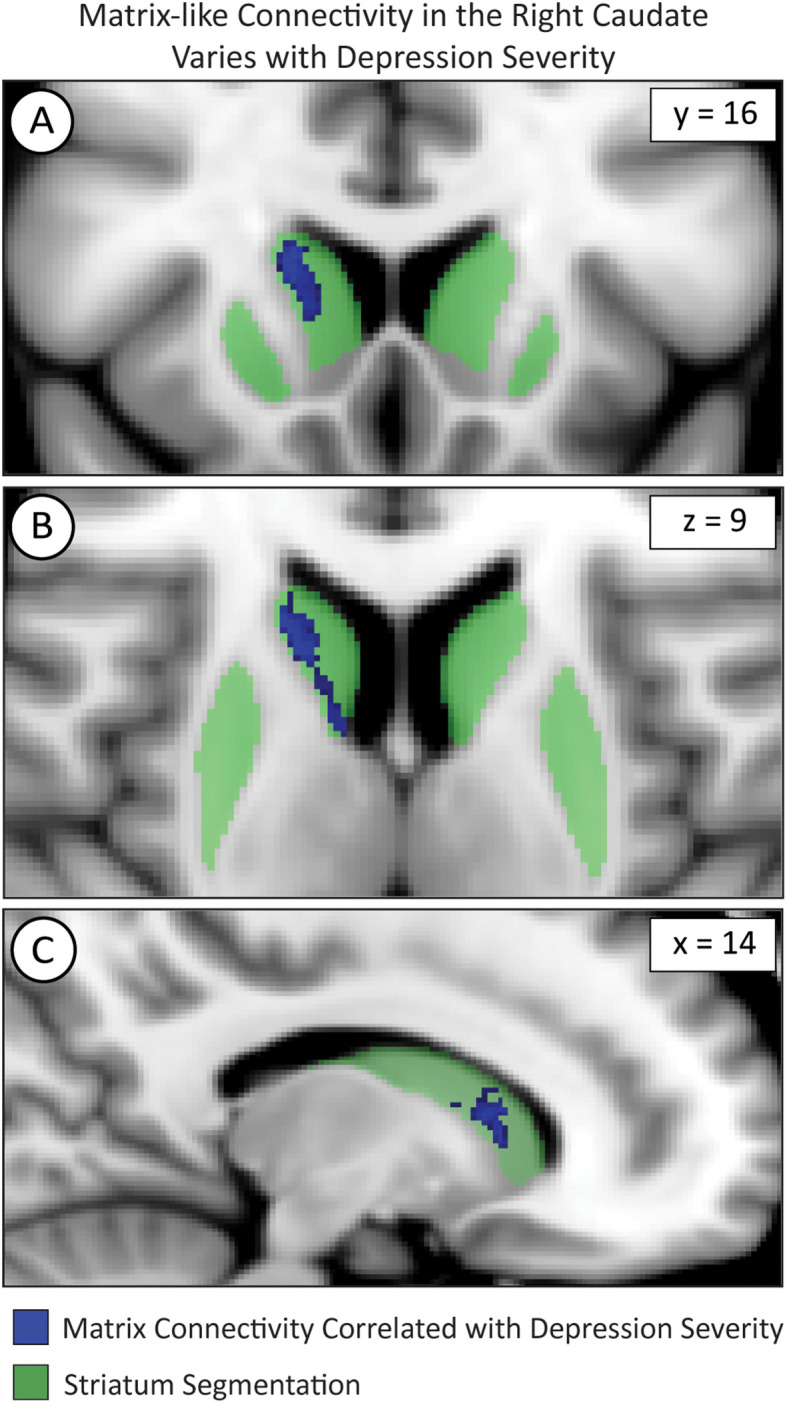
Voxelwise testing with FSL’s *randomise* identified voxel clusters whose matrix-like connectivity correlated with depression severity in the caudate as shown in the coronal **(A)**, axial **(B)**, and sagittal **(C)** planes. The images adhere to radiographic convention and coordinates follow MNI convention.

**Figure 5:**
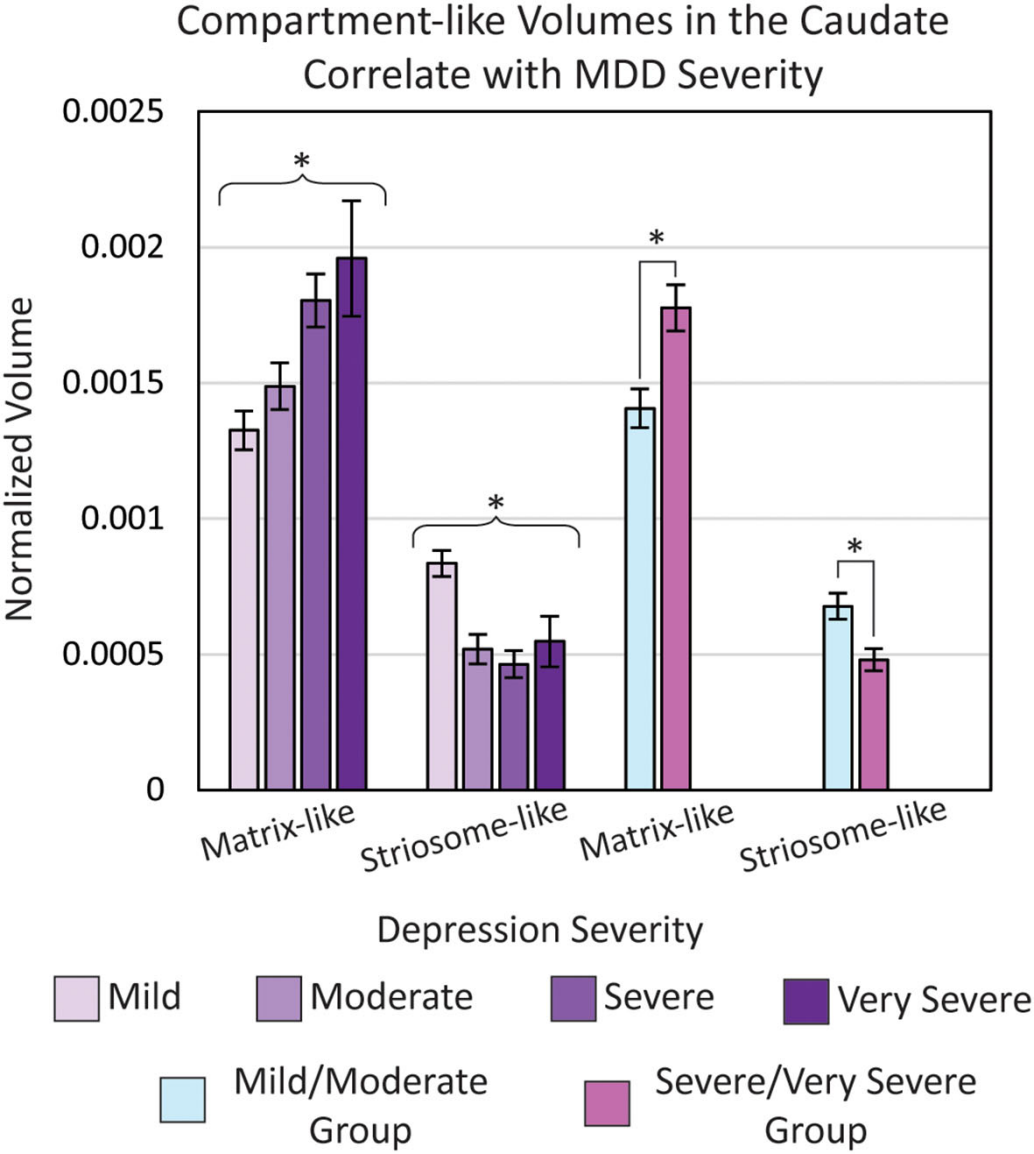
Normalized matrix-like (p = 0.043) and striosome-like (p = 4.9×10^-3^) volumes in the caudate were significantly correlated with major depressive disorder (MDD) severity (ANOVA, controlling for age, sex, and type of scanner). To examine the direction of this effect, we extracted volumes for each MDD severity level and compared volumes between a combined mild/moderate group and a combined severe/very severe group. The severe/very severe group showed a 26% increase in matrix-like volume (p = 1.2×10^-3^) and a 29% decrease in striosome-like volume (p = 1.1×10^-3^) relative to the mild/moderate group. Volumes were normalized to each subject’s total intracranial volume. *p< 0.05, FWE-corrected for multiple comparisons.

We used FSL *randomise* to test if compartment-like connectivity varied with depression severity (**Figure 4**). We identified a region in the right caudate where matrix-like bias was significantly correlated with depression severity (higher matrix-like volume in subjects with higher standardized depression scores). Both matrix-like (F_1,531_, p = 0.043) and striosome-like (F_1,531_, p = 4.9x10^-4^) volumes in the caudate were significantly correlated with MDD severity. Compartment-like volume in the putamen (**Figures 2**, **3**) did not correlate with MDD severity. We performed a *post-hoc* analysis, extracting compartment-like volumes in the caudate for each of our severity subgroups to identify the drivers of this group-level effect (**Figure 5**).

We compared eTIV-normalized compartment-like volumes in the whole-caudate between a combined mild/moderate severity group and a combined severe/very severe group. In the caudate, the severe/very severe group showed a 26% increase in matrix-like volume (p = 1.2x10^-3^) and a 29% decrease in striosome-like volume (p = 1.1x10^-3^) relative to the mild/moderate group. Raw compartment-like volume measures also exhibited this difference: Matrix (Mild/Moderate: 1936 mm^3^ vs. Severe/Very Severe: 2338 mm^3^; p = 1.4x10^-3^); Striosome (Mild/Moderate: 894 mm^3^ vs. Severe/Very Severe: 611 mm^3^; p = 8.0x10^-5^).

### 3.9 Somatotopic organization of corticostriate projections

**Figure 6:**
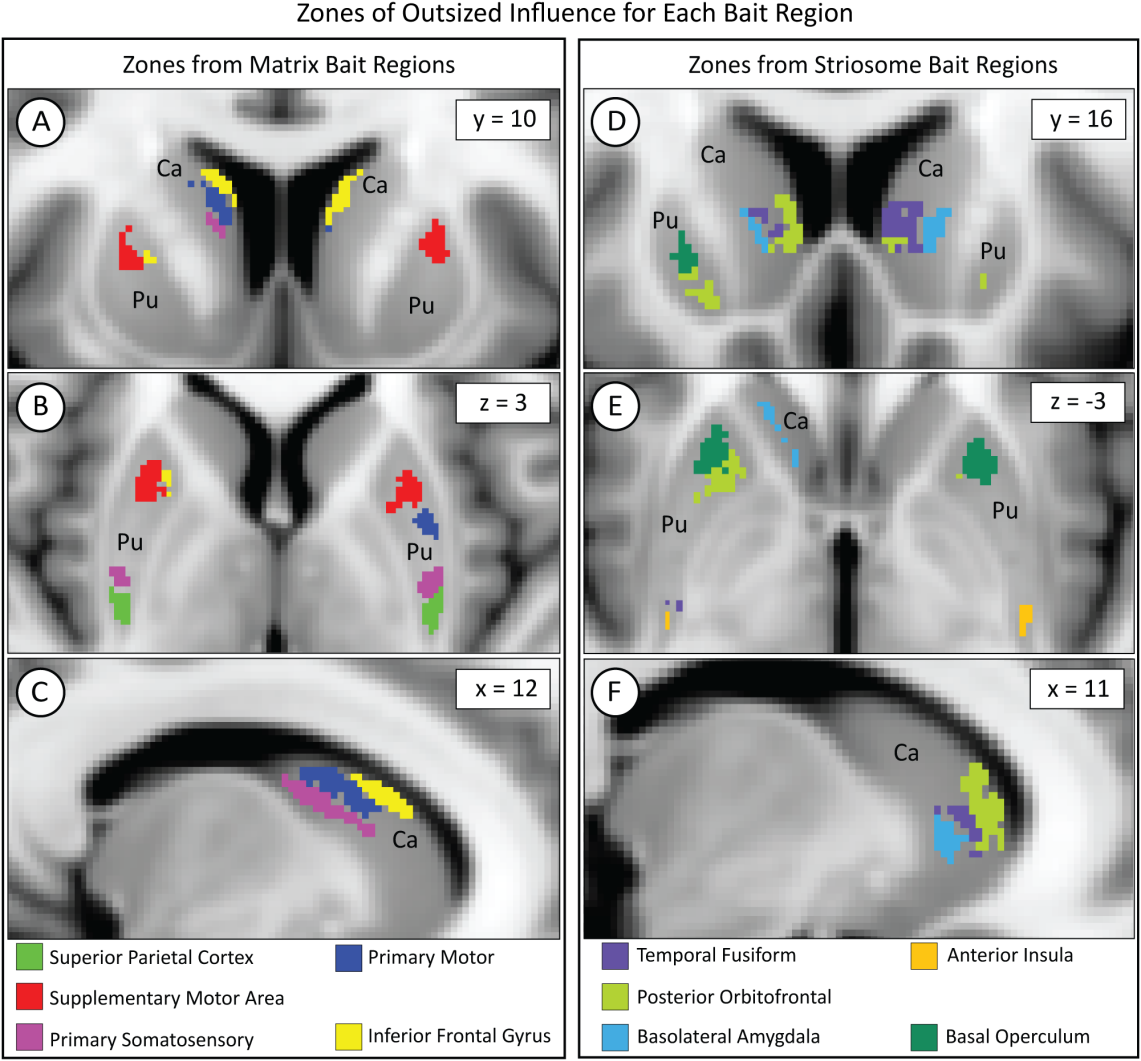
While each bait region projected broadly within the striatum, each region dominated the contribution to compartment-like bias within a discrete somatotopic zone, as seen in the coronal **(A, D)**, axial **(B, E)**, and sagittal **(C, F)** planes. Zones influenced by matrix-favoring bait regions are shown in **(A–C)**, while zones influenced by striosome-favoring bait regions are shown in **(D–F)**. Zones might border but never overlapped. Although we parcellated the left and right hemispheres independently, the location of each somatotopic zone was highly similar between hemispheres. Visible differences between the hemispheres (e.g., green voxels in **D**) are largely due to millimeter-scale differences in location (eg, green voxels in **E**). Pu, putamen; Ca, caudate. The images adhere to radiographic convention. Coordinates follow MNI convention.

We mapped the locations where each of our 10 bait regions had the greatest influence on compartment-like bias. To identify these somatotopic zones, we parcellated the striatum with nine out of our ten bait regions, leaving one bait region out. We then subtracted this “N-1” parcellation from the original striatal parcellation (which was based on all ten bait regions) to identify the influence of the left-out bait region. Although we parcellated the left and right hemispheres separately, the somatotopic zones attributable to each bait region were highly similar in size and location between hemispheres (**Figure 6**). Each somatotopic zone was spatially distinct with no overlap between zones. Notably, the somatotopic zones in these subjects are highly similar to the somatotopic zones we previously described in other cohorts (25, 40, 46).

**Figure 7:**
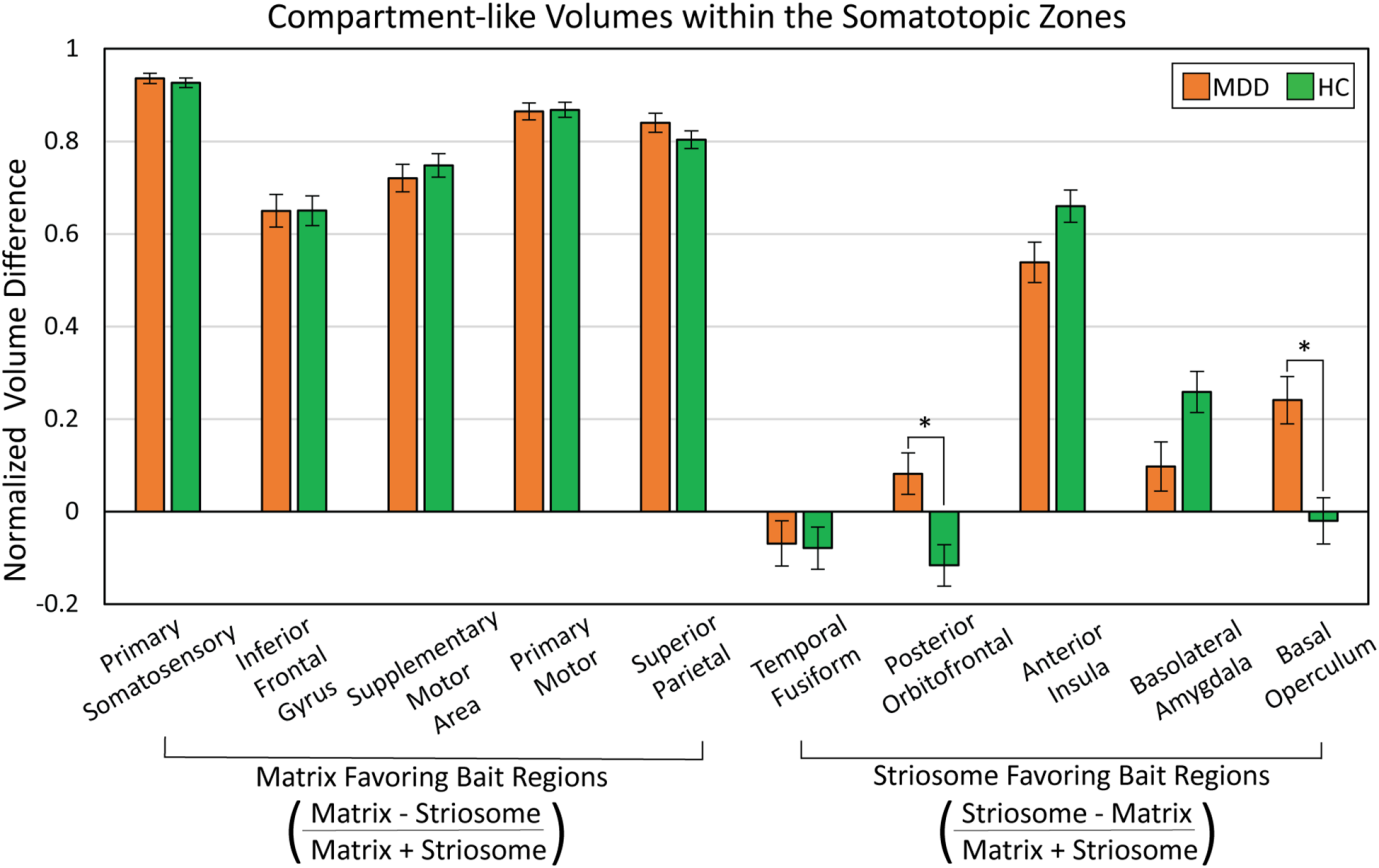
Abnormalities in compartment-like volumes were driven by particular bait regions. We assessed compartment-like volume within each somatotopic zone (see **Figure 6**), comparing major depressive disorder (MDD) and healthy control (HC) subjects. While somatotopic zones influenced by matrix-favoring bait regions (left sided bars) were always dominated by matrix-like volume (values >0), zones dominated by striosome-favoring bait regions (right sided bars) had a mix of striosome-dominated and near-neutral bias (values both above and below 0). MDD subjects had a shift from matrix-like volume to striosome-like volume in the zones dominated by posterior orbitofrontal cortex (p = 2.0x10^-4^) and basal operculum (p = 2.6x10^-4^). We expressed compartment-specific volume as the normalized ratio of volume difference $\left(\frac{Vol\left(Dominant\right)\;-\;Vol\left(Nondominant\right)}{Vol\left(Dominant\right)\;+\;Vol\left(Nondominant\right)}\right)$ . *p<0.015, FWE-corrected for multiple comparisons.suggests that reduced putamen volume in MDD correlates with a shift from matrix-based functional networks toward striosome-dominated patterns in the putamen.

We measured matrix-like and striosome-like volume (P≥0.55) within each somatotopic zone (**Figure 7**). We expressed these volume measures as a normalized ratio of volume change to capture changes in both compartments as a single value: $\left(\frac{Vol\left(Dominant\right)\;-\;Vol\left(Nondominant\right)}{Vol\left(Dominant\right)\;+\;Vol\left(Nondominant\right)}\right)$ . The dominant compartment refers to matrix-like volume for matrix-favoring bait regions and striosome-like volume for striosome-favoring bait regions.

In MDD, there were significant increases in matrix-like connectivity in the somatotopic zones dominated by the posterior orbitofrontal cortex (F_1,531_, p = 2.0x10^-4^) and basal operculum (F_1,531_, p = 2.6x10^-4^). Notably, these particular zones occupied the same area of rostral putamen in which our voxelwise assessment identified a shift toward striosome-like bias in MDD (compare **Figures 3** and **6**). It is important to note that each bait region also contributed to compartment-like bias in areas outside these somatotopic zones. However, these zones represent the area where a bait region’s contributions were stronger than those of all other regions.

## 4 Discussion

Subregions of cortex and striatum regulate each other through discrete cortico-striato-thalamo-cortical (CSTC) loops, an organizational schema that allows for diverging and converging information flows at multiple levels of processing. Given the central position of the striatum in these structural networks, compartment-specific abnormalities in the striatum can impact a broad range of brain functions (66). Matrix and striosome are embedded in segregated CSTC loops (23, 24) and are nodes in distinct resting-state functional connectivity networks (25). Shifting the ratio of matrix:striosome volume, or the strength of connectivity with one compartment, may influence the functions of extra-striate regions through these separated structural and functional networks. While the striatum has been extensively studied for its roles in habit formation, neurodegenerative diseases, drug addiction, and emotional regulation (37, 67), new regulatory functions for the striatum continue to be identified (68). In many mammalian species, including rats, mice, cats, dogs, non-human primates, and post-mortem human tissue, the organization of matrix and striosome (in terms of volume, location, and connectivity) is highly conserved (69–73). This nearly invariant organizational schema suggests that considerable evolutionary constraints have maintained distinct compartment-specific structure, and possibly, compartment-specific functions. Therefore, abnormalities in the abundance, location, or connectivity of matrix and striosome may impede normal brain functions, potentially influencing the cognitive, executive and limbic symptoms of MDD.

In our study, the total putamen volume was smaller in MDD, while caudate volume was similar between groups. In human and macaque tissue, the striosome in the putamen is organized into fewer but larger tubules than in the caudate (71). Tracer studies in squirrel monkeys demonstrated distinct and segregated patterns of connectivity between caudate and putamen, with each structure receiving input from largely separate neurons in the thalamus and midbrain while also projecting to different subregions of the globus pallidus and substantia nigra (74, 75). Anatomical and functional differences between the caudate and putamen may give rise to the nucleus-specific compartment abnormalities we observed in MDD.

Reduced putaminal volume is significantly correlated with increased risk of MDD (76). We found reduced putamen volume in our MDD group, as well as a shift from matrix-like to striosome-like volume in both eTIV normalized and raw measures in the putamen. The decreased putaminal size associated with MDD may be disproportionately affecting compartment volumes and subsequently, compartment function. This Voxelwise analysis of significant differences in compartment bias in the putamen revealed a bilaterally symmetric area in the rostral putamen in which bias was shifted from matrix-like toward striosome-like bias (**Figure 3**). In adolescents with severe depression (77), the rostral putamen had reduced resting-state functional connectivity with the superior frontal gyrus and supplementary motor area (a matrix-favoring region (78)). Our somatotopic zone analyses showed that the posterior orbitofrontal and basal operculum bait regions made outsized contributions to the abnormal volumes observed within the rostral putamen. The regions attributed to these zones aligned with the rostral putaminal region identified from the *randomise* comparison between MDD and HCs. The orbitofrontal cortex is involved in emotion and reward processing, and decreased functional activation of this region has been demonstrated in subjects with depression (79, 80). Similarly, in subjects with anxiety, the basal operculum is among the most-activated regions during the anticipation of threat (81). Abnormalities between the striatal compartments and these cortical regions may correlate with the underlying mechanisms of MDD.

Voxelwise testing in the caudate revealed an interhemispheric difference in compartment bias: the right hemisphere showed a large volume with shift toward matrix-like bias, while the left had a smaller, but significant shift toward striosome-like bias (**Figure 3**). Depression has been linked to hemispheric asymmetries using multiple neuroimaging modalities. For example, hemisphere-specific alterations in structural connectivity, such as right hemisphere caudate hypoconnectivity, has been reported in individuals with MDD (82). Other studies have also identified hemisphere-specific patterns of activation and activity in MDD, as observed in both EEG (83)and functional MRI (84). Unilateral brain lesions in the left cortex correlated with post-injury depression, while similar lesions in the right-hemisphere were associated with euphoria (85–88). Functional differences between the hemispheres may explain the lateralized compartment-specific alterations associated with MDD.

While there were no significant MDD vs. HC differences in whole-caudate or caudate compartment-like volumes, compartment-like volumes in the caudate varied with MDD severity (**Figure 5**). Notably, the shift from striosome-like to matrix-like volume in the caudate became more pronounced with increasing MDD severity. This pattern contrasts with the putamen, where we observed a shift in the opposite direction, from matrix-like to striosome-like volume. Such opposing compartmental changes between the caudate and putamen may point to a broader disruption in striatal compartmentalization, potentially reflecting abnormal embryologic migration or striatal development. Neuronal progenitors that will become striosome and matrix medium spiny neurons have distinct migratory and development patterns (89). Aberrant compartment development may lead to functional imbalances associated with the susceptibility to or severity of MDD.

Where diffusion MRI metrics such as fractional anisotropy (FA) and mean diffusivity (MD) have been traditionally used to assess white matter integrity, radial diffusivity is increasingly recognized as a valuable marker for microstructural differences in gray matter (90). FA, which measures the degree to which diffusivity is biased by tissue architecture, can have inconsistent measurements in gray matter due to the lack of organized fiber bundles. In contrast, RD measures diffusivity perpendicular to the dominant diffusion directions, which has been shown to be more consistent than FA or MD across gray matter regions within the same subject (91). For example, while FA may not accurately reflect myelin content in cortical gray matter, RD correlated with the underlying myelin and microstructural features in those sites (92). RD may offer greater sensitivity to structural abnormalities in gray matter structures with complex tissue architecture, a potential benefit for detecting changes in the matrix and striosome tissue compartments. Increased RD has been associated with abnormal tissue status, such as demyelination and axonal degeneration (93–95). We found no significant differences in RD between MDD and HC groups across whole nuclei or compartment-specific measures. This may suggest that the microstructural integrity of the nuclei and their compartments is preserved, indicating no overt loss of structural function. Compartment related abnormalities in MDD may therefore arise from factors other than local microstructural changes, such as imbalances in their relative influence on functional networks (25).

Striosomal neurons form direct inhibitory projections to dopamine neurons in the substantia nigra pars compacta, providing a mechanism by which the striatum can influence dopamine regulation, and consequently, reward-based learning and habits (43, 96). This architecture suggests that striosome abnormalities could directly alter nigral dopaminergic signaling. Moreover, the striosome compartment is selectively enriched with cannabinoid receptors (97), which regulate both dopaminergic firing patterns and emotional behaviors, including stress responses and social interaction (98, 99). Thus, the increase in striosome-like bias we identified in MDD, especially in the bilateral rostral putamen (**Figure 3**), may correlate with greater inhibition of dopamine release. Decreased and dysregulated dopaminergic release is associated with the symptoms of MDD (100–102), suggesting that excessive striosome-like function in the rostral putamen could produce the clinical features of depression.

Our findings have several important limitations. Connectivity-based striatal parcellation is based on probabilistic tractography, an inferential technique that cannot distinguish afferent from efferent projections, is susceptible to false positive and negative connectivity, and is substantially impacted by differing acquisition parameters, each of which can impact our measures of compartment-like bias (103). Another fundamental limitation of our striatal parcellation method is its reliance on injected tract tracers in animals as the gold standard for determining compartment-specific biases in structural connectivity. Connectivity may differ between animals and humans. Many brain areas that have been implicated in disorders of mood, motivation, and reward, such as MDD, have never been assessed through injected tract tracers. Some of these regions have no correlate in non-primate mammals, making it challenging to establish histologic ground truth. Another limitation is that the resolution of our diffusion voxels was larger than the maximum diameter of striosome branches (approximately 1.25 mm (27, 30)), suggesting that even highly biased striosome-like voxels will contain some matrix tissue. This limitation is likely compounded in obliquely sampled and smaller striosome branches, which may have led to loss of discrimination between the compartments. Higher-resolution diffusion MRI studies could distinguish matrix-like and striosome-like voxels with more granularity, potentially identifying voxels that included only matrix or only striosome tissue to enhance the accuracy of these findings. Notably, our method has a test-retest error of 0.14% (46) and the relative abundance, spatial distribution and extra-striate connectivity of compartment-like voxels match the anatomical features of matrix and striosome found in histology. However, while it is reassuring that compartment-like voxels match the anatomic features of the striatal compartments identified through histology, it is important for readers to recognize that our inferential method is not the equivalent of tissue-based identification of matrix and striosome.

Our MDD and healthy control cohorts were derived from four separate studies, each with different imaging resolutions and diffusion MRI acquisition protocols. Tractography results may be influenced by clinical factors, such as medication use among individuals with MDD (104). Information regarding medication use was not provided for most subjects, and thus we could not control for this potential confound. Comorbid psychiatric conditions which can be common in MDD populations may also affect streamline tractography results (105). Tractography may also be influenced by body mass index (BMI) (106), but BMI was not available for most subjects in this study. While we corrected our volumetric assessments for differences in head size (through normalization by eTIV), the inability to also control for BMI is a potential limitation of our tractographic analyses. Additionally, neurodevelopmental differences between adolescents and adults could affect white matter structure and diffusivity and thus alter tractography results. Since our combined dataset included both age groups, these developmental variations are a potential confound. To minimize the impact of scanner differences, sex or age on our results, we matched MDD and HC subjects within the same study, matched for sex and age, and also included scanner, sex, and age as regressors in our assessments of volume and diffusivity. However, the fact that our work blended subjects from multiple studies remains a limitation.

MDD is a disorder whose etiology is complex, combining genetic, social, and neurodevelopmental factors. This highlights the need for further research to identify the anatomic and neurophysiological abnormalities that may be related to depression. The striatum is implicated in motivation, executive function, motor control, and reward processing, each of which can be abnormal in MDD. However, due to challenges in obtaining histological samples for disease-specific analysis, the striatal compartments have not been adequately investigated in depression. Our findings suggest that the relative abundance of matrix and striosome may be altered in MDD, with distinct abnormalities in the caudate and putamen. Clarifying striatal compartment abnormalities in MDD deepens our understanding of the brain mechanisms that may contribute to this disabling and pervasive disorder.

## Data Availability

Publicly available datasets were analyzed in this study. This data can be found here: NIMH Data Archive data can be accessed at nda.nih.gov with study ID # 3090. Human Connectome Project data can be found here: https://www.humanconnectome.org/study/hcp-young-adult/document/1200-subjects-data-release The code, bait, seed, and exclusion masks necessary to complete striatal parcellation can be accessed here: github.com/jeff-waugh/Striatal-Connectivity-based-Parcellation.
